# Molecular Epidemiology of *Blastocystis* sp. in Various Animal Groups from Two French Zoos and Evaluation of Potential Zoonotic Risk

**DOI:** 10.1371/journal.pone.0169659

**Published:** 2017-01-06

**Authors:** Amandine Cian, Dima El Safadi, Marwan Osman, Romain Moriniere, Nausicaa Gantois, Sadia Benamrouz-Vanneste, Pilar Delgado-Viscogliosi, Karine Guyot, Luen-Luen Li, Sébastien Monchy, Christophe Noël, Philippe Poirier, Céline Nourrisson, Ivan Wawrzyniak, Frédéric Delbac, Stéphanie Bosc, Magali Chabé, Thierry Petit, Gabriela Certad, Eric Viscogliosi

**Affiliations:** 1 Université de Lille, CNRS, Inserm, CHU Lille, Institut Pasteur de Lille, U1019 –UMR 8204 –CIIL–Centre d’Infection et d’Immunité de Lille, Lille, France; 2 Laboratoire Microbiologie Santé et Environnement (LMSE), Ecole Doctorale des Sciences et de Technologie, Faculté de Santé Publique, Université Libanaise, Tripoli, Lebanon; 3 Parc Zoologique de Lille, Lille, France; 4 Laboratoire Ecologie et Biodiversité, Faculté Libre des Sciences et Technologies de Lille, Université Catholique de Lille, Lille, France; 5 Laboratoire d’Océanologie et de Géosciences, CNRS UMR 8187, Université du Littoral Côte d’Opale, Wimereux, France; 6 Geneius Laboratories Ltd., INEX Business Centre, Newcastle-upon-Tyne, United Kingdom; 7 Clermont Université, Université Blaise Pascal-Université d'Auvergne—CNRS UMR 6023 Laboratoire Microorganismes: Génome et Environnement, Clermont-Ferrand, France; 8 Parc Zoologique de La Palmyre, Les Mathes, France; 9 Département de la Recherche Médicale, Groupement des Hôpitaux de l'Institut Catholique de Lille, Faculté de Médecine et Maïeutique, Université Catholique de Lille, France; University of Ostrava, CZECH REPUBLIC

## Abstract

*Blastocystis* sp. is a common intestinal parasite infecting humans and a wide range of animals worldwide. It exhibits an extensive genetic diversity and 17 subtypes (STs) have thus far been identified in mammalian and avian hosts. Since several STs are common to humans and animals, it was proposed that a proportion of human infections may result from zoonotic transmission. However, the contribution of each animal source to human infection remains to be clarified. Therefore, the aim of this study was to expand our knowledge of the epidemiology and host specificity of this parasite by performing the largest epidemiological survey ever conducted in animal groups in terms of numbers of species screened. A total of 307 stool samples from 161 mammalian and non-mammalian species in two French zoos were screened by real-time PCR for the presence of *Blastocystis* sp. Overall, 32.2% of the animal samples and 37.9% of the species tested were shown to be infected with the parasite. A total of 111 animal *Blastocystis* sp. isolates were subtyped, and 11 of the 17 mammalian and avian STs as well as additional STs previously identified in reptiles and insects were found with a varying prevalence according to animal groups. These data were combined with those obtained from previous surveys to evaluate the potential risk of zoonotic transmission of *Blastocystis* sp. through the comparison of ST distribution between human and animal hosts. This suggests that non-human primates, artiodactyls and birds may serve as reservoirs for human infection, especially in animal handlers. In contrast, other mammals such as carnivores, and non-mammalian groups including reptiles and insects, do not seem to represent significant sources of *Blastocystis* sp. infection in humans. In further studies, more intensive sampling and screening of potential new animal hosts will reinforce these statements and expand our understanding of the circulation of *Blastocystis* sp. in animal and human populations.

## Introduction

*Blastocystis* sp. is an enteric protist with a worldwide distribution belonging to the group Stramenopiles and currently identified as one of the most common single-celled eukaryotes found in human stool samples [1–3]. Indeed, its prevalence can reach an average of 20% in industrialized countries [4,5] and can largely exceed 50% in developing countries [6]. A recent study even showed a prevalence of 100% in a cohort of children living in a rural area of Senegal [7]. Such a high prevalence of *Blastocystis* sp. clearly raises the question of the impact of this parasite in human health. Since asymptomatic carriage by *Blastocystis* sp. is very common, its role in human health and disease remains uncertain [8,9]. However, recent genomic data [10] coupled with *in-vitro* and *in-vivo* studies [11,12] allowed the identification of putative virulence factors and demonstrated the damaging effects of the parasite on the intestinal barrier, leading to plausible models of pathogenesis [8,13]. In addition, *Blastocystis* sp. colonization was shown to be associated with increased diversity of human gut bacterial microbiota [14], suggesting that the parasite requires high overall microbial diversity to become established in the human colon [3]. Much current data also suggests that this parasite should be associated with non-specific gastrointestinal symptoms including diarrhea, abdominal pain, and vomiting [1], and is suspected to be linked to irritable bowel syndrome [13] and urticaria [15].

*Blastocystis* sp. has also been reported in the intestinal tract of a wide range of animal hosts, including non-human primates (NHPs) and other mammals such as artiodactyls, perissodactyls, proboscideans, rodents, and marsupials, as well as birds, reptiles, amphibians, fish, annelids, and insects [16–18]. Among the genus *Blastocystis*, a large genetic diversity has been identified based on the comparison of small subunit (SSU) rDNA gene sequences. Consequently, isolates from mammalian and avian hosts were classified in 17 divergent lineages termed subtypes (STs) and arguably separate species [19]. Potential additional STs were also proposed in non-mammalian and avian hosts (so-called NMASTs in the present study for non-mammalian and avian STs) including amphibians, reptiles, and insects [20]. Among the 17 mammalian and avian STs, nine of them (ST1 to ST9) have been reported in humans with varying prevalence [2,21]. Approximately 90% of human isolates subtyped so far belong to ST1 to ST4, with a predominance of ST3 (around 60% of these isolates). With the exception of ST9 only found until now in humans, the other 8 STs display low to moderate host specificity by also colonizing various animal groups [6,19,20,22–26]. Consequently, these animal groups could represent potential reservoirs of zoonotic transmission, as reported in recent surveys [27–29]. Additional evidence strongly supports the zoonotic potential of *Blastocystis* sp., as a higher prevalence of this parasite is observed amongst animal handlers in comparison with individuals without contact with animals [24,25,30]. Moreover, successful experimental infections of chickens and rats with human isolates demonstrated the likely transmission of the parasite between human and animal hosts [8,31].

For a better understanding of the molecular epidemiology and transmission of *Blastocystis* sp., the ST distribution of the parasite was reported in several surveys conducted in a limited number of animal groups mainly from zoological gardens [19,23–25,32,33], national parks [34] and farms [30,35,36]. In France, only one study has recently been published on the identification and ST distribution of *Blastocystis* sp. in animals, which focused on a restricted cohort of household dogs [37]. Therefore, additional epidemiological data is needed to identify potential animal reservoirs of human infection. The first aim of the present study was thus to determine the prevalence of *Blastocystis* sp. among numerous animal groups in two French zoos, and to genetically characterize the positive samples in order to increase our knowledge of the epidemiology and host specificity of this parasite. The second goal was to compile our molecular data with all those available in the literature from previous epidemiological surveys in order to evaluate the potential risk of zoonotic transmission of each animal group through the comparative analysis of the ST distribution between human and animal populations.

## Materials and Methods

### Ethics statement

The study was conducted in the presence and under the responsibility of veterinarians from the two zoos. Only fecal samples collected after the spontaneous defecation of the zoo animals were analyzed. Consequently, this study did not require full Animal Ethics Committee approval in accordance with French law.

### Study sites

The Zoological Park of La Palmyre (La Palmyre Zoo) is located in the town of Les Mathes near Royan, in the department of Charente-Maritime, Southwest France, and is visited by nearly 700,000 people per year. It covers around 18 hectares and houses approximately 1,600 animals belonging to 115 different species. The Lille Zoo, located near the city center, in Northern France, hosts between 850,000 and 1 million visitors annually. It is nestled in a green area of 3.5 hectares and houses approximately 350 animals belonging to 70 different species.

### Sampling

At the La Palmyre Zoo, a total of 209 fresh fecal samples were collected in April 2014 from 94 different species, while a total of 98 fecal samples were obtained in June 2015 from 67 different species at the Lille Zoo. The full sampling covered a large variety of mammalian groups together with several avian orders and representatives of reptiles and insects. In both zoos, one to seven fecal samples were collected from each species screened, depending on the number of individuals housed by species. In addition, mixed and/or individual stool samples were obtained from each species, depending on whether the animals were housed separately or not. In the late afternoon, the majority of the animals in both zoos were moved from their day to night enclosures. Fresh fecal samples were thus collected early in the morning before the cleaning of animal cages. For some avian species with no night enclosures, collection of stool samples was also performed carefully directly on the ground or in their nests. For reptiles, feces were recovered from their pens while as fresh as possible. Collection of stool samples was performed in the presence of zookeepers and was strictly controlled to minimize potential contamination between animal species. Feces were collected in sterilized plastic containers using disposable spoons, then preserved at -20°C for further genomic investigation. At the Lille Zoo, house mice (*Mus musculus*) and Norway rats (*Rattus norvegicus*), as well as insects, were bred and given as food to some other animals. Mixed stool samples from these rodents were obtained in their cages after defecation and preserved as described above. Regarding insects, individuals were isolated in their flocks and then decapitated. The hindgut was immediately removed from the abdomen with a pair of tweezers and dissected into phosphate-buffered saline (PBS). The suspended content of the hindgut was aspirated and used for immediate DNA extraction. In the case of the desert locust, the droppings were also collected and analyzed.

### DNA extraction, amplification of the SSU rDNA gene and molecular subtyping of *Blastocystis* sp. isolates

Genomic DNA was extracted directly from approximately 250 mg of each fecal sample or from 100 μl of the hindgut content of insects in PBS using the QIAamp DNA Stool Mini Kit (Qiagen GmbH, Hilden, Germany) according to the manufacturer’s recommended procedures. DNA was eluted in 100 μl of AE buffer (Qiagen) to increase its concentration and stored at -20°C until being analyzed. For each sample, 1 μl of extracted DNA was subjected to a real-time Polymerase Chain Reaction (qPCR) assay using the *Blastocystis* sp.-specific primers BL18SPPF1 (5’-AGTAGTCATACGCTCGTCTCAAA-3’) and BL18SR2PP (5’-TCTTCGTTACCCGTTACTGC-3’) designed by Poirier et al. [38]. These primers target a DNA fragment of 320 to 342 bp of the *Blastocystis* sp. SSU rDNA gene, depending on the ST. This domain of the SSU-rDNA gene has been shown to provide sufficient information for differentiating STs of *Blastocystis* sp. [5,38]. DNA extraction controls (isolation of DNAs without stool and from a *Blastocystis* sp.-negative stool) subsequently used in qPCR assays and positive (DNA obtained from *Blastocystis* sp. ST4 or ST7 cultures) and negative (DNA matrix replaced by water) qPCR controls were performed. The positive qPCR products were purified and directly sequenced on both strands in a sequencing facility (Genoscreen, Lille, France). Direct sequencing of several qPCR products generated mixed signals that could reflect infections by different STs. These samples were thus re-analyzed by non-qPCR using the same primer pair as for qPCR. Non-qPCR amplifications were performed in 50 μl according to standard conditions for Platinum *Taq* High-Fidelity DNA polymerase (Invitrogen, Groningen, the Netherlands). After denaturation at 94°C for 5 min, 40 cycles of amplification were performed with a Bioer LifeECO apparatus (Binjiang District, China) as follows: 30 s at 94°C, 35 s at 60°C, and 50 s at 68°C. The final extension was continued for 2 min. Non-qPCR products were separated by agarose gel electrophoresis and bands of the expected size (approximately 320 bp) were purified using the Wizard SV Gel and PCR clean-up system (Promega, Madison, WI, USA). Purified PCR products were cloned in the T-vector, pCR 2.1-TOPO (Invitrogen) and amplified in *Escherichia coli* One Shot TOP10 competent cells. Minipreparations of plasmid DNA were done using the NucleoSpin Plasmid kit (Macherey-Nagel, Düren, Germany). Five positive clones containing inserts of approximately the expected size were arbitrarily selected for each sample and sequenced on both strands. The SSU rDNA gene sequences obtained in this study were deposited in GenBank under accession numbers KR259402 to KR259512 (S1 Table). The sequences obtained were compared with all *Blastocystis* sp. homologous sequences available from the National Centre for Biotechnology Information (NCBI) using the nucleotide BLAST program. The STs were identified by determining the exact match or closest similarity against all known mammalian and avian *Blastocystis* sp. STs according to the last classification by Alfellani et al. [19].

### Phylogenetic analysis of *Blastocystis* spp. isolates

Nine of the SSU rDNA gene sequences obtained in the present study for eight *Blastocystis* sp. isolates exhibited low similarity (≤ 92%) with homologous sequences available in the databases. To clarify their identification via phylogenetic tools, these sequences were thus added to a large dataset including (i) 33 sequences of *Blastocystis* sp. isolates representing ST1 to ST10 and ST13 to ST17 (ST11 and ST12 SSU rDNA gene sequences are not yet available for this particular domain) [25] and (ii) 22 homologous sequences representing 7 potential NMASTs according to the recent phylogenetic analysis by Yoshikawa et al. [20]. The SSU rDNA genes sequences were aligned using the BioEdit v7.2.5 package (www.mbio.ncsu.edu/BioEdit/bioedit.html*)*. All positions containing gaps were eliminated and the phylogenetic inference was restricted to 246 sites that could be unambiguously aligned. Phylogenetic analyses were performed using Neighbor-Joining and maximum likelihood methods implemented in MEGA6. The maximum likelihood analysis was based on the Tamura-Nei model [39], and initial trees for the heuristic search were obtained by applying the Neighbor-Joining method to a matrix of pairwise distances estimated using the Maximum Composite Likelihood (MCL) approach. Bootstrap proportions (BPs) were obtained from 1,000 pseudo-replicates. Bayesian Posterior Probabilities (BPPs) were calculated from 1,000 replicates using MrBayes 3.2.6 with the maximum likelihood method for 10 million generations.

## Results and Discussion

### Overall prevalence and ST diversity of *Blastocystis* sp. in two French zoos

A total of 307 individual or mixed animal samples (Table 1) were collected in both French zoos from NHPs, Carnivora, Artiodactyla, Perissodactyla, Proboscidea, Rodentia, Chiroptera, Marsupialia, Aves, Crocodilia, Squamata, Testudines, and Insecta, then screened for the presence of *Blastocystis* sp. by qPCR. This sampling represented 161 species, only 18 of which were housed in both zoos, and covered a wide variety of mammalian and non-mammalian groups. At the time of this survey, fecal samples were obtained from 82% and 96% of animal species hosted in the La Palmyre and Lille zoos respectively. Of the 209 samples and 94 species tested at the La Palmyre Zoo, 75 (35.9%) and 41 (43.6%) respectively were positive for *Blastocystis* sp. At the Lille Zoo, the prevalence of the parasite was 24.5% (24/98) and 29.9% (20/67) among the samples and species screened respectively. Overall, 32.2% (99/307) of the animal samples and 37.9% (61/161) of the species tested in the present study were shown to be infected with *Blastocystis* sp. By comparison, such a high prevalence of the parasite had been previously observed in two smaller-scale molecular studies conducted in Perth Zoo, Australia, with a different sampling of species. In the first Australian study, 68.2% of the 22 samples and 69.2% of the 13 species analyzed were positive for *Blastocystis* sp. [23]. This prevalence was slightly lower (52.3% of the 120 samples and 55.6% of the 36 species screened) in the second Australian study conducted more recently [25]. Still in Australia, 9/55 (16.4%) samples and 4/12 (33.3%) species tested at Taronga Zoo were identified via PCR as positive for the parasite [33]. Together, these molecular surveys all clearly show that *Blastocystis* sp. is an extremely common parasite colonizing a wide range of animal hosts kept in captivity in zoological gardens. However, this was not the conclusion of an epidemiological study conducted at a zoo of Kuala Lumpur, Malaysia, in which a total of 197 stool samples from 16 species of primates, 21 species of hoofed mammals and 9 feline species were tested for the presence of intestinal parasites, with only 2 samples collected from 2 primate species being described as positive for *Blastocystis* sp. [40]. To explain the low prevalence of the parasite in the Malaysian zoo, it should be emphasized that the identification of the parasite was performed using direct-light microscopy of fecal smears and formol ether concentration techniques, both methods being shown to be far less sensitive than PCR [38]. Moreover, the sensitivity of microscopy diagnosis is highly dependent on the experience of the observer and the optimization of the experimental protocol [4].

**Table 1:** Animal samples collected from various hosts at the French zoos in Lille and La Palmyre. According to Groves [41], the Cebidae includes the ancient family of Callitrichidae (marmosets and tamarins).

Common name and systematics	Scientific name	Zoos^a^	Sample numbers^b^	Type of samples^c^	Sequence positive samples for *Blastocystis* sp.
***EUTHERIA***					
**PRIMATES**					
**Catarrhini**					
**Hominidae**					
Western lowland gorilla	*Gorilla gorilla*	LP	6	3 IS + 3 MS	4
Orangutan	*Pongo pygmaeus*	LP	3	3IS	3
Chimpanzee	*Pan troglodytes*	LP	3	1 IS + 2 MS	2
**Hylobatidae**					
Lar gibbon	*Hylobates lar*	ZL	2	2 MS	2
Lar gibbon	*Hylobates lar*	LP	1	1 MS	0
Buff-cheeked gibbon	*Nomascus gabriellae*	LP	2	2 MS	2
Siamang	*Symphalangus syndactylus*	ZL	4	1 IS + 3 MS	4
**Cercopithecidae**					
Southern pig-tailed macaque	*Macaca nemestrina*	LP	3	3 MS	3
Mandrill	*Mandrillus sphinx*	LP	1	1 MS	1
Owl-faced monkey	*Cercopithecus hamlyni*	LP	2	1 IS + 1 MS	1
Roloway monkey	*Cercopithecus roloway*	LP	1	1 MS	1
L’Hoest’s monkey	*Cercopithecus lhoesti*	LP	1	1 MS	1
De Brazza’s monkey	*Cercopithecus neglectus*	LP	2	2 MS	2
Kikuyu black-and-white colobus	*Colobus guereza*	LP	3	1 IS + 2 MS	2
**Platyrrhini**					
**Cebidae**					
Brown capuchin	*Cebus apella*	ZL	1	1 MS	0
Buff-headed capuchin	*Cebus xanthosternos*	LP	2	2 MS	0
Common squirrel monkey	*Saimiri sciureus*	LP	1	1 MS	0
Emperor tamarin	*Saguinus imperator*	ZL	1	1 MS	0
Emperor tamarin	*Saguinus imperator*	LP	2	2 MS	1
White-lipped tamarin	*Saguinus labiatus*	ZL	1	1 MS	0
Red-handed tamarin	*Saguinus midas*	LP	2	2 MS	0
Pied tamarin	*Saguinus bicolor*	LP	1	1 MS	0
Cotton-top tamarin	*Saguinus oedipus*	LP	1	1 MS	0
Golden-headed lion tamarin	*Leontopithecus chrysomelas*	LP	4	4 MS	3
Golden lion tamarin	*Leontopithecus rosalia*	LP	1	1 MS	0
Geoffroy's marmoset	*Callithrix geoffroyi*	ZL	1	1 MS	0
Geoffroy's marmoset	*Callithrix geoffroyi*	LP	1	1 MS	0
Pigmy marmoset	*Callithrix pygmaea*	LP	2	2 MS	0
Common marmoset	*Callithrix jacchus*	LP	1	1 MS	0
Goeldi's marmoset	*Callimico goeldii*	LP	2	2 MS	0
**Pitheciidae**					
White-faced saki	*Pithecia pithecia*	ZL	1	1 MS	1
**Strepsirrhini**					
**Lemuridae**					
Ring-tailed lemur	*Lemur catta*	ZL	1	1 MS	1
Ring-tailed lemur	*Lemur catta*	LP	4	4 MS	4
Red ruffed lemur	*Varecia rubra*	ZL	1	1 MS	1
Red ruffed lemur	*Varecia rubra*	LP	2	1 IS + 1 MS	2
Black-and-white ruffed lemur	*Varecia variegata*	ZL	1	1 MS	1
Black-and-white ruffed lemur	*Varecia variegata*	LP	3	1 IS + 2 MS	1
Blue-eyed black lemur	*Eulemur flavifrons*	LP	1	1 MS	1
**Lorisidae**					
Pygmy slow loris	*Nycticebus pygmaeus*	ZL	1	1 MS	0
**CARNIVORA**					
**Feliformia**					
**Felidae**					
Northern lynx	*Lynx lynx*	LP	1	1 IS	0
Lion	*Panthera leo*	LP	1	1 IS	0
Cheetah	*Acinonyx jubatus*	LP	5	2 IS + 3 MS	1
Leopard	*Panthera pardus*	LP	2	2 IS	0
Snow leopard	*Panthera uncia*	LP	2	2 IS	0
Jaguar	*Panthera onca*	LP	2	2 IS	0
Tiger	*Panthera tigris*	LP	2	2 MS	0
**Viverridae**					
Binturong	*Arctictis binturong*	ZL	1	1 IS	0
**Herpestidae**					
Yellow mongoose	*Cynictis penicillata*	ZL	1	1 MS	0
Slender-tailed meerkat	*Suricata suricatta*	ZL	1	1 MS	0
Slender-tailed meerkat	*Suricata suricatta*	LP	2	2 IS	0
**Caniformia**					
**Canidae**					
Grey wolf	*Canis lupus*	LP	4	4 IS	1
African hunting dog	*Lycaon pictus*	LP	3	3 IS	0
Fennec fox	*Vulpes zerda*	LP	7	2 IS + 5 MS	0
**Ailuridae**					
Red panda	*Ailurus fulgens*	ZL	2	2 IS	0
Red panda	*Ailurus fulgens*	LP	2	2 IS	0
**Ursidae**					
Polar bear	*Ursus maritimus*	LP	3	3 IS	1
**Mustelidae**					
Oriental small-clawed otter	*Aonyx cinerea*	LP	1	1 MS	0
**Procyonidae**					
Kinkajou	*Potos flavus*	ZL	1	1 IS	0
Brown-nosed coati	*Nasua nasua*	ZL	1	1 MS	0
Brown-nosed coati	*Nasua nasua*	LP	3	2 IS + 1 MS	0
**Otariidae**					
California sealion	*Zalophus californianus*	LP	6	6 IS	1
**ARTIODACTYLA**					
**Camelidae**					
Alpaca	*Vicugna pacos*	ZL	1	1 MS	1
Alpaca	*Vicugna pacos*	LP	5	5 MS	0
**Hippopotamidae**					
Hippopotamus	*Hippopotamus amphibius*	LP	1	1 IS	0
**Giraffidae**					
Giraffe	*Giraffa camelopardalis*	LP	6	6 IS	4
**Tragulidae**					
Java mouse-deer	*Tragulus javanicus*	ZL	1	1 MS	1
**Bovidae**					
Common eland	*Taurotragus oryx*	ZL	1	1 IS	1
Greater kudu	*Tragelaphus strepsiceros*	LP	2	2 MS	2
Bongo	*Tragelaphus eurycerus*	LP	1	1 MS	1
American bison	*Bison bison*	LP	3	3 IS	1
Blindled wildebeest	*Connochaetes taurinus*	LP	1	1 MS	1
Blesbok	*Damaliscus pygargus phillipsi*	LP	1	1 MS	0
Beisa oryx	*Oryx beisa*	LP	4	4 MS	3
Scimitar-horned oryx	*Oryx dammah*	LP	5	5 IS	5
Goat	*Capra hircus*	LP	7	7 MS	1
Impala	*Aepyceros melampus*	LP	1	1 MS	0
**PERISSODACTYLA**					
**Equidae**					
Common zebra	*Equus burchellii*	ZL	2	1 IS + 1 MS	1
Common zebra	*Equus burchellii*	LP	2	2 IS	1
Grevy’s zebra	*Equus grevyi*	LP	2	2 MS	0
Poitou donkey	*Equus asinus*	LP	2	2 MS	2
**Rhinocerotidae**					
White rhinoceros	*Ceratotherium simum*	ZL	2	2 IS	0
White rhinoceros	*Ceratotherium simum*	LP	1	1 MS	0
**Tapiridae**					
South American tapir	*Tapirus terrestris*	ZL	1	1 IS	1
South American tapir	*Tapirus terrestris*	LP	2	2 IS	2
**PROBOSCIDEA**					
Asiatic elephant	*Elephas maximus*	LP	4	4 IS	3
**RODENTIA**					
House mouse^d^	*Mus musculus*	ZL	2	2 MS	0
Norway rat^d^	*Rattus norvegicus*	ZL	2	2 MS	1
Indian crested porcupine	*Hystrix indica*	ZL	2	2 IS	0
Patagonian mara	*Dolichotis patagonum*	ZL	3	3 MS	0
Capybara	*Hydrochoerus hydrochaeris*	ZL	2	1 IS + 1 MS	1
Capybara	*Hydrochoerus hydrochaeris*	LP	3	2 IS + 1 MS	2
**CHIROPTERA**					
Lyle's flying fox	*Pteropus lylei*	ZL	2	1 IS + 1 MS	0
Rodrigues flying fox	*Pteropus rodricensis*	LP	1	1 MS	1
Egyptian fruit bat	*Rousettus aegyptiacus*	LP	1	1 MS	1
***MARSUPIALIA***					
Red kangaroo	*Macropus rufus*	LP	1	1 MS	0
Red-necked wallaby	*Macropus rufogriseus*	LP	1	1 MS	1
***AVES***					
**Galliformes**					
Crested wood partridge	*Rollulus rouloul*	ZL	1	1 MS	0
Common peafowl	*Pavo cristatus*	LP	2	2 IS	1
**Anseriformes**					
Bar-headed goose	*Anser indicus*	ZL	1	1 IS	0
Bar-headed goose	*Anser indicus*	LP	1	1 MS	0
Barnacle goose	*Branta leucopsis*	ZL	1	1 IS	0
Nene	*Branta sandvicensis*	ZL	2	2 IS	0
Mandarin duck	*Aix galericulata*	ZL	1	1 MS	0
Tufted duck	*Aythya fuligula*	ZL	1	1 MS	0
Ferriginous duck	*Aythya nyroca*	ZL	3	3 MS	0
Hottentot teal	*Anas hottentota*	ZL	1	1 MS	0
Black swan	*Cygnus atratus*	LP	1	1 MS	0
**Psittaciformes**					
Twenty-eight parrot	*Barnardius zonarius*	ZL	1	1 MS	0
Grey parrot	*Psittacus erithacus*	ZL	1	1 MS	0
Senegal parrot	*Poicephalus senegalus*	ZL	1	1 MS	0
Burrowing parrot	*Cyanoliseus patagonus*	ZL	1	1 MS	0
Green-winged macaw	*Ara chloroptera*	ZL	1	1 MS	0
Green-winged macaw	*Ara chloroptera*	LP	4	4 MS	0
Buffon’s macaw	*Ara ambigua*	LP	1	1 MS	0
Scarlet macaw	*Ara macao*	ZL	1	1 MS	0
Blue-and-yellow macaw	*Ara ararauna*	LP	4	4 MS	0
Hyacinth macaw	*Anodorhynchus hyacinthinus*	LP	1	1 MS	0
Blue-crowned conure	*Aratinga acuticaudata*	ZL	1	1 MS	0
Monk parakeet	*Myiopsitta monachus*	ZL	1	1 MS	0
Orange-winged amazon	*Amazona amazonica*	ZL	1	1 MS	0
Mealy amazon	*Amazona farinosa*	ZL	1	1 MS	0
Yellow-crowned amazon	*Amazona ochrocephala*	ZL	1	1 MS	0
Rosella	*Platycercus eximus*	LP	1	1 MS	0
Lesser sulphur-crested cockatoo	*Cacatua sulphurea*	LP	1	1 MS	0
**Strigiformes**					
Snowy owl	*Bubo scandiacus*	ZL	1	1 MS	0
**Coraciiformes**					
Laughing kookaburra	*Dacelo novaeguineae*	ZL	2	2 MS	0
**Passeriformes**					
Javan sparrow	*Lonchura oryzivora*	ZL	1	1 MS	0
**Phoenicopteriformes**					
Chilean flamingo	*Phoenicopterus chilensis*	LP	6	1 IS + 5 MS	0
American flamingo	*Phoenicopterus ruber*	LP	3	3 MS	1
**Pelecaliformes**					
Scarlet ibis	*Eudocimus ruber*	LP	4	4 MS	0
**Accipitriformes**					
Rüppel’s griffon vulture	*Gyps rueppellii*	LP	1	1 MS	0
**Ciconiiformes**					
Marabou stork	*Leptoptilos crumeniferus*	LP	1	1 MS	0
**Columbiformes**					
Nicobar pigeon	*Caloenas nicobarica*	LP	1	1 MS	0
**Bucerotiformes**					
Southern ground-hornbill	*Bucorvus leadbeateri*	LP	1	1 IS	0
Trumpeter hornbill	*Bycanistes bucinator*	LP	1	1 MS	0
Great Indian hornbill	*Buceros bicornis*	LP	1	1 MS	0
**Passeriformes**					
Bali mynah	*Leucopsar rotschildi*	LP	1	1 IS	0
**Gruiformes**					
Grey crowned-crane	*Balearica regulorum*	LP	2	2 IS	0
**Sphenisciformes**					
Jackass penguin	*Spheniscus demersus*	LP	1	1 MS	0
**Ratites**					
Common ostrich	*Struthio camelus*	LP	2	2 MS	2
Greater rhea	*Rhea americana*	LP	3	3 IS	2
***CROCODILIA***					
African slender-snouted crocodile	*Mecistops cataphractus*	LP	1	1 IS	0
***SQUAMATA***					
Green iguana	*Iguana iguana*	ZL	1	1 IS	1
Green iguana	*Iguana iguana*	LP	1	1 IS	0
Green anaconda	*Eunectes murinus*	LP	1	1 IS	0
Boa constrictor	*Boa constrictor*	ZL	1	1 IS	1
Cornsnake	*Pantherophis guttatus*	ZL	2	2 MS	0
Russian ratsnake	*Elaphe schrencki*	ZL	1	1 IS	0
Taiwan beauty snake	*Elaphe taeniura*	ZL	1	1 IS	0
Common kingsnake	*Lampropeltis getula*	ZL	2	2 IS	0
Milksnake	*Lampropeltis triangulum*	ZL	2	2 IS	0
***TESTUDINATA***					
Aldabra tortoise	*Aldabrachelys gigantea*	ZL	1	1 IS	1
Aldabra tortoise	*Aldabrachelys gigantea*	LP	1	1 IS	0
Spur-thighed tortoise	*Testudo graeca*	ZL	1	1 IS	1
***INSECTA***^***d***^					
Madagascar hissing cockroach	*Gromphadorhina portentosa*	ZL	3	3 IS	0
Giant cockroach	*Blaberus giganteus*	ZL	1	1 IS	1
Peppered roach	*Archimandrita tessellata*	ZL	1	1 IS	0
Dubia roach	*Blaptica dubia*	ZL	3	3 IS	1
Sun beetle	*Pachnoda marginata*	ZL	2	2 IS	0
Desert locust	*Schistocerca gregaria*	ZL	2	2 IS	1
Field cricket	*Gryllus bimaculatus*	ZL	5	5 IS	0

^a^ ZL: Zoo of Lille; LP: Zoo of La Palmyre

^b^ Depending on the species, the samples were obtained either from a single individual or a group of individuals of the same species or both. In the case of insects, samples analyzed were the gastrointestinal tract of single individuals obtained after dissection. Only droppings of the desert locust were obtained and analyzed in addition to that of the gastrointestinal tract

^c^ IS: Individual sample; MS: Mixed sample

^d^ Animals bred in the Lille Zoo and used as food for other animals

Of the 99 samples positive for *Blastocystis* sp. by qPCR from the two French zoos, 88 represented infections by a single ST according to the resulting sequence chromatograms. In the remaining 11 qPCR products, chromatogram analysis revealed the presence of double signals, suggesting mixed infections by different STs. Cloning of the non-qPCR product obtained from each of these 11 samples was then necessary for subtyping, and 5 positive clones were arbitrarily selected and sequenced for each cloning. Ten of the 11 samples showed mixed infection with two different STs, whereas the remaining sample harbored 3 different STs. Therefore, with the addition of 11 mixed infections consisting of either two or three different STs, a total of 111 *Blastocystis* sp. isolates were subtyped and analyzed in this study. 102 of the corresponding 111 SSU rDNA gene sequences showed 96% to 100% identity to representative sequences of the 17 mammalian and avian *Blastocystis* sp. STs reported so far [19], allowing the direct subtyping of these isolates (Table 2). Of these 17 STs, 11 (ST1, ST2, ST3, ST4, ST5, ST7, ST8, ST10, ST13, ST14 and ST15) were identified, with varying prevalence between animal groups as detailed below, highlighting the large genetic diversity observed between *Blastocystis* sp. isolates from animals. To a lesser extent, a wide range of mammalian and avian STs was also previously described in several smaller-scale surveys conducted in zoological gardens [19,24,25,33].

**Table 2:** Subtype results from sequence-positive samples for *Blastocystis* sp.

Host	Zoos^a^	Sequences	*Blastocystis* sp. STs																		Untypable
			1	2	3	4	5	6	7	8	9	10	11	12	13	14	15	16	17	NMASTs^b^	
***EUTHERIA***																					
**PRIMATES**																					
Western lowland gorilla	LP	4	1	1	-	-	2	-	-	-	-	-	-	-	-	-	-	-	-	-	-
Orangutan	LP	3	-	-	-	-	3	-	-	-	-	-	-	-	-	-	-	-	-	-	-
Chimpanzee	LP	2	-	1	-	-	1	-	-	-	-	-	-	-	-	-	-	-	-	-	-
Lar gibbon	ZL	2	-	1	-	-	-	-	-	-	-	-	-	-	-	-	1	-	-	-	-
Siamang	ZL	4	-	-	3	1	-	-	-	-	-	-	-	-	-	-	-	-	-	-	-
Buff-cheeked gibbon	LP	2	2	-	-	-	-	-	-	-	-	-	-	-	-	-	-	-	-	-	-
Southern pig-tailed macaque	LP	4	3	-	1	-	-	-	-	-	-	-	-	-	-	-	-	-	-	-	-
Mandrill	LP	1	1	-	-	-	-	-	-	-	-	-	-	-	-	-	-	-	-	-	-
Owl-faced monkey	LP	1	1	-	-	-	-	-	-	-	-	-	-	-	-	-	-	-	-	-	-
Roloway monkey	LP	1	-	1	-	-	-	-	-	-	-	-	-	-	-	-	-	-	-	-	-
L’Hoest’s monkey	LP	1	-	-	1	-	-	-	-	-	-	-	-	-	-	-	-	-	-	-	-
De Brazza’s monkey	LP	3	1	-	2	-	-	-	-	-	-	-	-	-	-	-	-	-	-	-	-
Kikuyu black-and-white colobus	LP	2	1	1	-	-	-	-	-	-	-	-	-	-	-	-	-	-	-	-	-
Emperor tamarin	LP	1	-	-	1	-	-	-	-	-	-	-	-	-	-	-	-	-	-	-	-
Golden-headed lion tamarin	LP	3	3	-	-	-	-	-	-	-	-	-	-	-	-	-	-	-	-	-	-
White-faced saki	ZL	1	-	1	-	-	-	-	-	-	-	-	-	-	-	-	-	-	-	-	-
Ring-tailed lemur	LP	4	1	3	-	-	-	-	-	-	-	-	-	-	-	-	-	-	-	-	-
Ring-tailed lemur	ZL	1	-	-	-	-	-	-	-	1	-	-	-	-	-	-	-	-	-	-	-
Red ruffed lemur	LP	4	1	-	-	1	1	-	-	1	-	-	-	-	-	-	-	-	-	-	-
Red ruffed lemur	ZL	1	-	-	-	-	-	-	1	-	-	-	-	-	-	-	-	-	-	-	-
Black-and-white ruffed lemur	LP	1	-	-	-	1	-	-	-	-	-	-	-	-	-	-	-	-	-	-	-
Black-and-white ruffed lemur	ZL	1	-	-	-	-	-	-	1	-	-	-	-	-	-	-	-	-	-	-	-
Blue-eyed black lemur	LP	1	-	-	-	1	-	-	-	-	-	-	-	-	-	-	-	-	-	-	-
**Total**		**48**	**15**	**9**	**8**	**4**	**7**	**-**	**2**	**2**	**-**	**-**	**-**	**-**	**-**	**-**	**1**	**-**	**-**	**-**	**-**
**CARNIVORA**																					
Cheetah	LP	1	-	1	-	-	-	-	-	-	-	-	-	-	-	-	-	-	-	-	-
Grey wolf	LP	1	-	-	1	-	-	-	-	-	-	-	-	-	-	-	-	-	-	-	-
Polar bear	LP	1	-	-	1	-	-	-	-	-	-	-	-	-	-	-	-	-	-	-	-
California sea lion	LP	1	1	-	-	-	-	-	-	-	-	-	-	-	-	-	-	-	-	-	-
**Total**		**4**	**1**	**1**	**2**	**-**	**-**	**-**	**-**	**-**	**-**	**-**	**-**	**-**	**-**	**-**	**-**	**-**	**-**	**-**	**-**
**ARTIODACTYLA**																					
Alpaca	ZL	1	-	-	-	-	-	-	-	-	-	1	-	-	-	-	-	-	-	-	-
Java mouse-deer	ZL	1	-	-	-	-	-	-	-	-	-	-	-	-	1	-	-	-	-	-	-
Giraffe	LP	5	-	-	-	-	-	-	-	-	-	1	-	-	-	4	-	-	-	-	-
Common eland	ZL	1	-	-	-	-	-	-	-	-	-	-	-	-	-	1	-	-	-	-	-
Greater kudu	LP	2	-	-	-	-	-	-	-	-	-	2	-	-	-	-	-	-	-	-	-
Bongo	LP	1	-	-	-	-	-	-	-	-	-	1	-	-	-	-	-	-	-	-	-
American bison	LP	2	-	-	-	-	-	-	-	-	-	1	-	-	-	1	-	-	-	-	-
Blindled wildebeest	LP	1	-	-	-	-	-	-	-	-	-	-	-	-	-	1	-	-	-	-	-
Beisa oryx	LP	3	-	-	-	-	-	-	-	-	-	3	-	-	-	-	-	-	-	-	-
Scimitar-horned oryx	LP	5	-	-	1	-	-	-	-	-	-	4	-	-	-	-	-	-	-	-	-
Goat	LP	2	-	-	-	-	-	-	-	-	-	1	-	-	-	1	-	-	-	-	-
**Total**		**24**	**-**	**-**	**1**	**-**	**-**	**-**	**-**	**-**	**-**	**14**	**-**	**-**	**1**	**8**	**-**	**-**	**-**	**-**	**-**
**PERISSODACTYLA**																					
Common zebra	LP	1	-	-	1	-	-	-	-	-	-	-	-	-	-	-	-	-	-	-	-
Common zebra	ZL	1	-	-	-	-	-	-	-	-	-	-	-	-	-	-	-	-	-	-	1
Poitou donkey	LP	3	-	1	2	-	-	-	-	-	-	-	-	-	-	-	-	-	-	-	-
South American tapir	LP	2	-	-	2	-	-	-	-	-	-	-	-	-	-	-	-	-	-	-	-
South American tapir	ZL	1	-	-	-	-	1	-	-	-	-	-	-	-	-	-	-	-	-	-	-
**Total**		**8**	**-**	**1**	**5**	**-**	**1**	**-**	**-**	**-**	**-**	**-**	**-**	**-**	**-**	**-**	**-**	**-**	**-**	**-**	**1**
**PROBOSCIDEA**																					
Asiatic elephant	LP	5	3	-	2	-	-	-	-	-	-	-	-	-	-	-	-	-	-	-	-
**Total**		**5**	**3**	**-**	**2**	**-**	**-**	**-**	**-**	**-**	**-**	**-**	**-**	**-**	**-**	**-**	**-**	**-**	**-**	**-**	
**RODENTIA**																					
Capybara	LP	2	-	1	-	-	-	-	-	-	-	-	-	-	-	-	-	-	-	-	1
Capybara	ZL	1	-	-	-	-	1	-	-	-	-	-	-	-	-	-	-	-	-	-	-
Norway rat	ZL	1	-	-	-	1	-	-	-	-	-	-	-	-	-	-	-	-	-	-	-
**Total**		**4**	**-**	**1**	**-**	**1**	**1**	**-**	**-**	**-**	**-**	**-**	**-**	**-**	**-**	**-**	**-**	**-**	**-**	**-**	**1**
**CHIROPTERA**																					
Rodrigues flying fox	LP	1	-	-	1	-	-	-	-	-	-	-	-	-	-	-	-	-	-	-	-
Egyptian fruit bat	LP	1	-	-	1	-	-	-	-	-	-	-	-	-	-	-	-	-	-	-	-
**Total**		**2**	**-**	**-**	**2**	**-**	**-**	**-**	**-**	**-**	**-**	**-**	**-**	**-**	**-**	**-**	**-**	**-**	**-**	**-**	**-**
***MARSUPIALIA***																					
Red-necked wallaby	LP	1	-	-	-	-	-	-	-	-	-	-	-	-	-	-	-	-	-	-	1
**Total**		**1**	**-**	**-**	**-**	**-**	**-**	**-**	**-**	**-**	**-**	**-**	**-**	**-**	**-**	**-**	**-**	**-**	**-**	**-**	**1**
***AVES***																					
Common peafowl	LP	1	-	-	-	-	-	-	-	-	-	-	-	-	-	-	-	-	-	-	1
American flamingo	LP	1	1	-	-	-	-	-	-	-	-	-	-	-	-	-	-	-	-	-	-
Common ostrich	LP	2	-	-	-	-	2	-	-	-	-	-	-	-	-	-	-	-	-	-	-
Greater rhea	LP	3	-	-	-	2	1	-	-	-	-	-	-	-	-	-	-	-	-	-	-
**Total**		**7**	**1**	**-**	**-**	**2**	**3**	**-**	**-**	**-**	**-**	**-**	**-**	**-**	**-**	**-**	**-**	**-**	**-**	**-**	**1**
***SQUAMATA***																					
Green iguana	ZL	1	-	-	-	-	-	-	-	-	-	-	-	-	-	-	-	-	-	-	1
Boa constrictor	ZL	1	-	-	-	-	-	-	-	-	-	-	-	-	-	-	-	-	-	-	1
**Total**		**2**	**-**	**-**	**-**	**-**	**-**	**-**	**-**	**-**	**-**	**-**	**-**	**-**	**-**	**-**	**-**	**-**	**-**	**-**	**2**
***TESTUDINATA***																					
Aldabra tortoise	ZL	1	-	-	-	-	-	-	-	-	-	-	-	-	-	-	-	-	-	1	-
Spur-thighed tortoise	ZL	2	-	-	-	-	-	-	-	-	-	-	-	-	-	-	-	-	-	2	-
**Total**		**3**	**-**	**-**	**-**	**-**	**-**	**-**	**-**	**-**	**-**	**-**	**-**	**-**	**-**	**-**	**-**	**-**	**-**	**3**	**-**
***INSECTA***																					
Giant cockroach	ZL	1	-	1	-	-	-	-	-	-	-	-	-	-	-	-	-	-	-	-	-
Dubia roach	ZL	1	-	-	1	-	-	-	-	-	-	-	-	-	-	-	-	-	-	-	-
Desert locust	ZL	1	-	1	-	-	-	-	-	-	-	-	-	-	-	-	-	-	-	-	-
**Total**		**3**	**-**	**2**	**1**	**-**	**-**	**-**	**-**	**-**	**-**	**-**	**-**	**-**	**-**	**-**	**-**	**-**	**-**	**-**	**-**
**Grand total**		**111**	**20**	**14**	**21**	**7**	**12**	**0**	**2**	**2**	**0**	**14**	**0**	**0**	**1**	**8**	**1**	**0**	**0**	**3**	**6**

^a^ ZL: Zoo of Lille; LP: Zoo of La Palmyre

^b^ Non Mammalian and Avian STs

**Fig 1:**
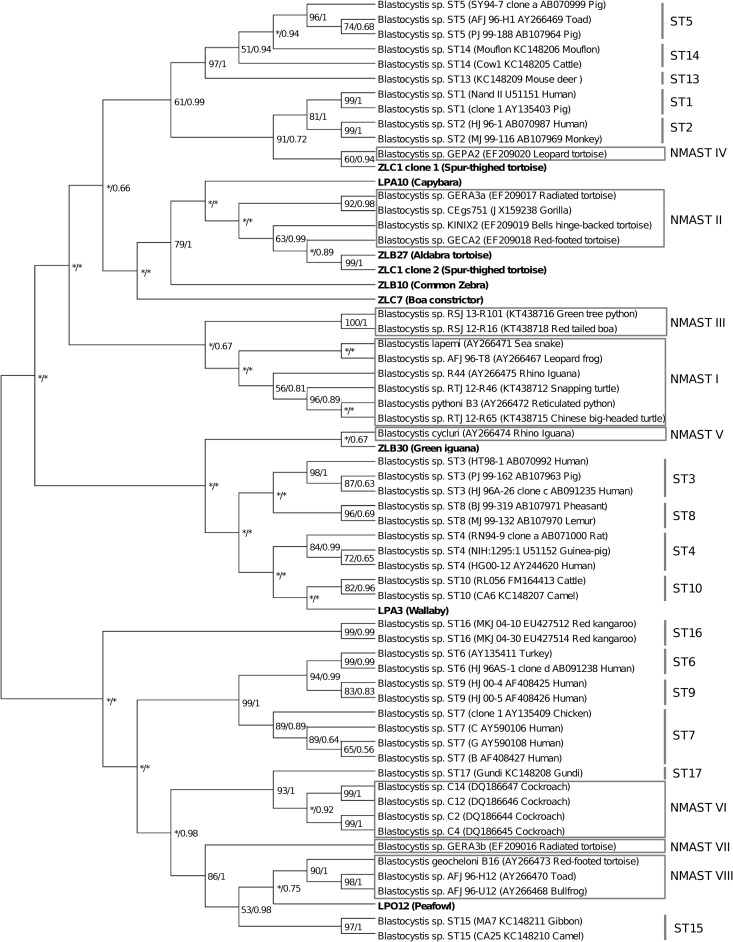
Unrooted maximum likelihood cladogram based on SSU rDNA gene sequences depicting relationships between *Blastocystis* sp. isolates. The numbers at the node indicate BPs and BPPs respectively, given by ML and Bayesian analyses with 1,000 replicates. The asterisks designate nodes with BPs or BPPs below 50% or 0.5. The sequences obtained in this study are shown in bold.

The remaining nine SSU rDNA gene sequences obtained in the present study from eight *Blastocystis* sp. isolates exhibited 82% to 92% identity with homologous sequences available in databases, thus preventing direct subtyping of the corresponding isolates. Consequently, these sequences were added to an existing database including 55 homologous sequences from representatives of both mammalian/avian STs and so-called NMASTs, then applied to a phylogenetic analysis in order to clarify their relationships (Fig 1). With the exception of ST13 and ST17 (only one representative isolate), all mammalian and avian STs were represented by 2 to 4 isolates. In the ML *Blastocystis* sp. tree, almost all mammalian and avian STs included in the analysis formed monophyletic lineages strongly supported by BP and BPP values. Only the two ST14 isolates represented a paraphyletic clade. Interestingly, even if this tree was reconstructed from short-length SSU rDNA gene sequences, some groupings were consistent with those described in previous phylogenetic analyses based on full-length sequences of the same gene [20]. This was the case of two large clades uniting with strong robustness ST1, ST2, ST5, ST13 and ST14 (BP and BPP of 61% and 0.99 respectively) as well as ST6, ST7 and ST9 (BP and BPP of 99% and 1 respectively). ST3, ST4, ST8 and ST10 also grouped together, but this clustering was not supported by BP and BPP values (below 50% and 0.5 respectively). The 22 isolates obtained from non-mammalian and avian hosts in previous studies and included in the phylogenetic reconstruction were shown to exhibit a large genetic diversity and are thus classified in eight clades called NMAST I to NMAST VIII, three of which (NMASTs IV, V and VII) are represented by only a single isolate. Three of these clades (NMASTs I, II and III) were recently proposed as potential novel STs [20]. NMASTs I to V and NMAST VII represented reptilian clusters, while NMAST VI was composed only of insect isolates and NMAST VIII of both amphibian and reptilian isolates. Curiously, an additional isolate identified through the microbiota analysis of a wild western lowland gorilla [42] emerged within the reptilian NMAST II, suggesting highly probable accidental contamination of the primate with reptile feces in its natural environment. Concerning the 9 sequences obtained in the present study and exhibiting low identity with the homologous sequences available in databases, 2 of them, ZLB27 from aldabra tortoise and ZLC1 clone 2 from spur-thighed tortoise, grouped together (BP and BPP of 99% and 1, respectively) and clustered with two representatives of the NMAST II clade (KINIX2 and GECA2) with BP and BPP of 63% and 0.99, respectively. This significant support suggested that ZLB27 and ZLC1 clone 2 sequences could be assigned to the reptilian NMAST II clade. Regarding the second sequence obtained from spur-thighed tortoise (ZLC1 clone 1), its clustering with the ZLB30 sequence from green iguana which currently represents the only representative of the reptilian NMAST IV was well supported (BP and BPP of 60% and 0.94 respectively) and consequently, the ZLC1 clone 1 sequence was assigned to the NMAST IV clade. In contrast, the emergence of the 6 other sequences obtained in the present study remained to be clarified. Indeed, the LPA10, ZLB10 and ZLC7 sequences from capybara, common zebra and boa constrictor, respectively, formed sister groups with the reptilian NMAST II sequences but with low BP and BPP supports. In addition, the clustering with various clades of ZLB30, LPA3 and LPO12 sequences from green iguana, wallaby and peafowl, respectively, was not supported by BP and BPP values. Therefore, pending further phylogenetic analyses of longer SSU rRNA gene sequences to clarify the emergence of these 6 isolates, all have for now been assigned as “untypable”.

### Prevalence of *Blastocystis* sp. in mammalian groups housed in French zoos

In our epidemiological survey, NHPs were the most represented animal group, with a total of 73 samples collected in the two French zoos (Table 1). These samples covered 39 species (with 6 species common to both zoos) belonging to 7 NHPs families. Strikingly, 60.3% (44/73) of the NHP samples and 59.0% (23/39) of the NHP species tested in both French zoos were positive for *Blastocystis* sp. Such a high prevalence of the parasite was also previously reported in different NHP families in various countries and habitats. Limiting ourselves to the most recent literature focusing on NHP cohorts of significant size, the prevalence of *Blastocystis* sp. reached 87.6% in a population of 443 captive cynomolgus macaques (Cercopithecidae) housed in biomedical research centers in Italy [43], 60.4% in a group of 96 mantled howler monkeys (Atelidae) from a reserve in Ecuador [44], and 51.3% and 49% in wild chimpanzee (Hominidae) communities living in the savanna area of Ugalla in Tanzania [45] and the Cantanhez National Park in Guinea-Bissau [46] respectively. A high prevalence of the parasite was also observed in large groups of chimpanzees (71.4%), but also vervet (84.7%) and colobus (83.7%) monkeys (Cercopithecidae), all three species living on Rodondo Island in Tanzania [34]. Interestingly, more than half of the primate species housed in Perth Zoo in Australia [25], around 92% of the NHP species tested at the Osaka city zoo in Japan [47], and numerous captive primate species from animal facilities, zoos and sanctuaries in various countries [24,32] were also shown to be infected with *Blastocystis* sp. From all these data, and as confirmed in the present study, NHPs clearly represent common hosts of the parasite, regardless of their lifestyle and geographical origin.

The group of Carnivora was widely sampled in the present study (Table 1). However, only 4 of the 53 samples (7.5%) tested and obtained from 22 species (with 3 species common to both zoos) were positive for *Blastocystis* sp. by qPCR. The four species (18.2%) infected with the parasite, namely cheetah, grey wolf, polar bear and California sealion, were all housed at the La Palmyre Zoo and, to our knowledge, had never yet been described as hosts of *Blastocystis* sp. Such low parasite prevalence in carnivores living in captivity was previously observed, for instance, at the Osaka city zoo in Japan where none of the 11 species tested were infected [47]. The parasite was also absent in the 7 carnivore species of different geographical origins investigated by Alfellani et al. [19]. Until now, epidemiological surveys of Carnivora focused mostly on Canidae and more generally on various dog populations from different countries. In the large majority of published surveys, canine fecal samples were either found to be negative for *Blastocystis* sp. [23,47–49] or infected by the parasite with a low or moderate prevalence ranging between 1.3% and 14.5% [37,50–54]. However, in some studies mainly focused on stray dogs living mostly in areas of poor sanitation and hygiene, the prevalence of *Blastocystis* sp. was significantly higher [6,51].

In the animal groups studied in the present survey, Artiodactyla have also been extensively sampled, since 40 samples were collected from 15 species (with 1 species common to both zoos) belonging to 5 families (Table 1). In numerous epidemiological studies including artiodactyls, sampling was mainly focused on domestic Bovidae and Suidae living in farms or zoological gardens in close contact with humans and whose feces could easily be collected from a large number of individuals [6,19,23–26,28,30,33,35,36,47,55–57]. Of the 40 samples and 15 species screened in our study from artiodactyls, 21 (52.5%) and 11 (73.3%) respectively were positive for *Blastocystis* sp. by qPCR. Interestingly, 8 of the 10 bovid species tested (80%) were shown to be infected by the parasite, which was in agreement with previous studies in which *Blastocystis* sp. were mentioned as a common parasite in Bovidae. Indeed, the prevalence of the parasite reached 71% and 80% in cattle in Japan [47] and Colombia [6] respectively, and was over 30% in goats in Malaysia [55]. The highly endemic status of *Blastocystis* sp. in Artiodactyla was also confirmed with a prevalence of 87.1% and 95% observed in cohorts of domestic pigs in Indonesia [57] and Japan [47] respectively. In addition, 76.7% and 46.8% of pigs from commercial intensive piggeries in Australia [30] and Spain [35], together with 45.2% of pigs from a village in rural Cambodia [30], were positive for the parasite.

Regarding other mammalian groups included in the present study, the number of samples collected was more limited than for the NHPs, Carnivora and Artiodactyla. Indeed, a total of 14 samples were collected from Perissodactyla, representing 8 species (3 of which were common to both zoos), 4 samples from Proboscidea representing a single species (Asian elephant), 14 samples from Rodentia (6 species represented, one of which was common to both zoos), and 4 samples from 3 species of Chiroptera (Table 1). The prevalence of species infected with *Blastocystis* sp. in these animal groups was very high and reached 62.5% (5/8 species) in perissodactyls, 100% (1/1) in proboscideans, 50% (3/6) in rodents, and 66.7% (2/3) in bats. Very few epidemiological surveys have been conducted to date among these 4 animal groups, as summarized by Alfellani et al. [19], and consequently, any comparison in term of the prevalence between cohorts of the same animal group remains insignificant.

Finally, two species (red kangaroo and red-necked wallaby) belonging to the mammalian infraclass Marsupialia, both housed at the La Palmyre Zoo, were also screened for the presence of *Blastocystis* sp. Only one of the two (red-necked wallaby) (50%) was shown to be positive for the parasite. In comparison, 62.5% and 52.6% of the samples obtained from opossums in Colombia [6] and 7 species of marsupials in Australia [33] respectively were infected by *Blastocystis* sp.

### Prevalence of *Blastocystis* sp. in non-mammalian groups housed in French zoos

In the present survey, birds were the most widely represented among non-mammalian animals, with a total of 70 individual or mixed animal samples collected in the two French zoos (Table 1). These samples represented 45 species (with 2 species common to both zoos) belonging to 16 avian orders. Within this broad and highly diversified sampling, the overall prevalence of *Blastocystis* sp. in birds was very low at 8.6% (6/70) of the samples and 8.9% (4/45) of the species tested were positive for the parasite. Only one galliform (peafowl), one phoenicopteriform (American flamingo), and two ratite (ostrich and greater rhea) species were thus shown to be infected by *Blastocystis* sp. and were all housed at the La Palmyre Zoo. No positive cases were identified in birds at the Lille Zoo. In term of prevalence of the parasite, these data strongly contrast with almost all previous surveys focused on birds. Indeed, studies conducted in Japanese zoos [45,58] and including pheasants, ducks, chickens and ostriches, revealed a very high prevalence of *Blastocystis* sp. ranging from 56% to 100%, depending on the avian group tested. Similarly, 90% of the samples screened from 5 wild bird species in Colombia were shown to be infected by *Blastocystis* sp. [6]. The parasite was also present in 75% to 100% of domestic chickens, ducks, and geese, and commercially farmed ostrich samples collected in Australia [59], and in approximately 95% of chickens reared on 4 Australian farms [60]. The prevalence of the parasite also reached 34% in a cohort of domestic chickens in an Indonesian community [57], and 32.9%, 46.6%, and 23.9% in chickens, ducks and quails respectively, sold at municipal markets in Brazil [61], and 58% and 100% of ostriches screened on European [62] and Malaysian [63] commercial farms respectively. It remains difficult to compare the avian parasite prevalence observed in the present study with that of previous surveys, since they were conducted on different populations of zoo, wild or farmed birds, in various geographical areas and using different detection methods. However, strict hygiene conditions coupled with individual housing of the majority of bird species in both French zoos likely explains the limitation of *Blastocystis* sp. infection in this animal group.

Another large group of vertebrates analyzed in this study was that of reptiles, including the three orders Crocodilia, Squamata, and Testudinata. A total of 16 reptilian samples were collected in the two French zoos from 13 species (with 2 species common to both zoos) (Table 1). *Blastocystis* sp. was not detected in Crocodilia, but was found in Squamata and Testudinata in 2/12 (16.7%) and 2/3 (66.7%) respectively of the samples tested, suggesting that the parasite is common in reptiles. Previous studies reinforce this hypothesis, since the prevalence of *Blastocystis* sp. was shown to be 28.6% among 28 species of reptiles housed at the Singapore zoological gardens [64] and 7% among house lizards captured in Kuala Lumpur, Malaysia [65]. Moreover, although this group of animals cannot be sampled easily, parasite isolates were also characterized in various reptilian species [66–68].

Finally, the last group of animals sampled in this study was that of insects, all housed at the Lille Zoo. A total of 17 samples were obtained from 7 different species (Table 1), and three of them (42.9%) were shown to be positive for *Blastocystis* sp. The first study describing the parasite and its prevalence in insects was by Zaman et al. [69], who identified *Blastocystis* sp. in 80% of 10 cockroaches belonging to the species *Periplaneta americana* and caught from sewage tanks in Singapore. A lower prevalence (10%) of the parasite was found by Suresh et al. [65] in a group of 30 individuals of the same cockroach species, but caught in dwellings in Malaysia. Additional isolates were also obtained from *P*. *americana* by Yoshikawa et al. [68]. On the other hand, *Blastocystis* sp. was unsuccessfully identified in houseflies belonging to the insect order Diptera [65].

### ST distribution of *Blastocystis* sp. in animal groups and risk assessment of zoonotic transmission

The first findings of the large-scale study described above have expanded our knowledge on the prevalence of *Blastocystis* sp. in a variety of animal groups. Through the subsequent molecular identification of all these animal isolates, this survey could also help evaluate the zoonotic potential of the parasite. Indeed, overlapping of STs, coupled with necessary high similarity or even identity of SSU rDNA gene sequences between human and animal isolates, highlights the transmission between hosts as reported in recent surveys [27–29]. In several recent epidemiological studies [6,32], alleles were assigned to SSU rDNA gene sequences spanning the barcode region designed by Scicluna et al. [73] and compared between human and animal isolates to investigate zoonotic transmission. Because of its length, this longer domain of the SSU rDNA gene (around 600 bp) is not applicable to the qPCR assay used in the present study that requires a routine screening of a large number of samples. Consequently, the SSU rDNA region sequenced in our survey did not cover the entire domain necessary for allele assignment.

**Fig 2:**
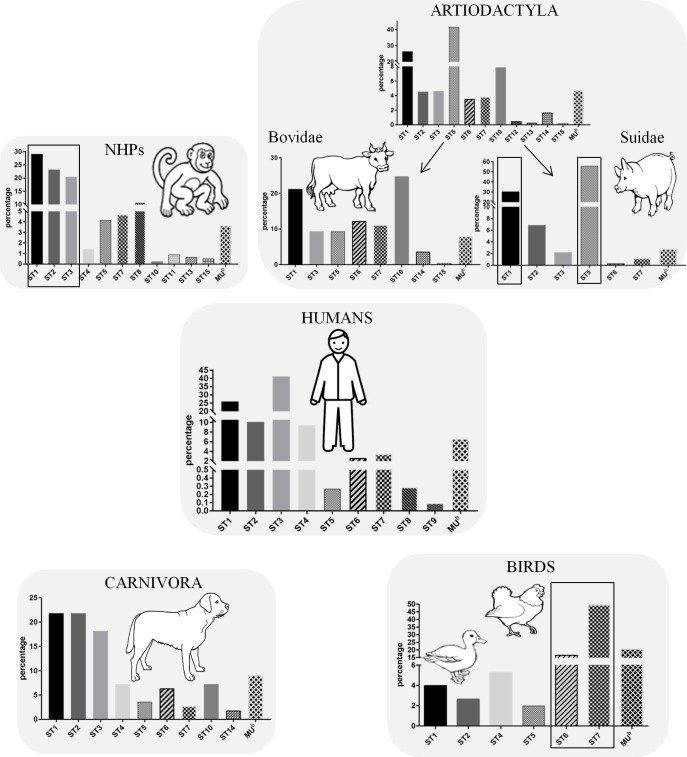
Comparison of the distribution and relative prevalence of *Blastocystis* sp. STs in humans and major animal groups and evaluation of potential zoonotic risk. Histograms showing the prevalence of each ST as a percentage were obtained from the total values summarized in Table 3 for humans and the four most sampled animal groups, including NHPs, artiodactyls, carnivores, and birds. In the case of artiodactyls, the data were presented globally for this animal group, but also separately for Bovidae and Suidae. The predominant STs found in these animal groups that could likely be transmitted to humans are boxed. MU = Mixed ST / untypable.

Among the 44 NHPs samples that were sequence-positive for *Blastocystis* sp. by qPCR, 3 sequences traces clearly represented mixed ST infections. Indeed, two different STs colonized southern pig-tailed macaque and De Brazza’s monkey, and three STs infected red ruffed lemur. These three primate species were all housed at the La Palmyre Zoo. Therefore, a total of 48 isolates were subtyped from NHP samples (Table 2) and the corresponding sequences belonged to ST1 (31.2%), ST2 (18.7%), ST3 (16.7%), ST4 (8.3%), ST5 (14.6%), ST7 (4.2%), ST8 (4.2%) and ST15 (2.1%). This ST distribution was roughly similar to that compiled from all molecular data currently available from captive and wild NHPs (Table 3), especially with a large predominance of ST1 to ST3. Indeed, these three STs represented around 67% of the isolates identified in NHPs in the present study. The only minor differences compared to the compiled data were the higher prevalence of ST4 and ST5 and the lower prevalence of both ST7 and ST8 in our survey. As previously stated by Alfellani et al. [32], the concordance in the distribution of STs across epidemiological studies conducted in NHPs suggest strongly that predominant ST1 to ST3 are shared by captive and wild monkeys and thus naturally occur in this host group. Interestingly, the evolutionarily closely related NHP and human groups share a high prevalence of the parasite and the same predominance of ST1 to ST3 among isolates. Moreover, 32 of the 48 (66.7%) sequences of isolates belonging to ST1 to ST3 identified from NHPs in the present study were shown to be 100% identical to homologous sequences available in databases from humans. Similarly, the sequences of one of the four ST4 isolates and of the two ST8 isolates from NHPs also exhibited 100% identity with those of humans. In contrast, the sequences of NHP isolates belonging to ST7 and ST5 were 98 and 97% similar respectively compared to those from humans. Therefore, the genetic identity of ST1 to ST4 and ST8 isolates from NHPs and humans, together with the high prevalence rate of *Blastocystis* sp. in primates, strongly suggests that NHPs might serve as a reservoir for human infection, in particular for ST1 to ST3 (Fig 2). To reinforce this statement, the zoonotic potential of NHP isolates was previously demonstrated by Yoshikawa et al. [27], in which rhesus monkeys and children living in the same area of Nepal shared ST2 isolates with identical sequences. For these authors, poor sanitary infrastructure might cause the contamination of the food and water supply by NHP feces. In another study, Stensvold et al. [24] showed the frequent infection of primate handlers by ST8 in the United Kingdom, while this ST is rarely found in humans but is common in NHPs. Daily contact with NHP feces in the course of their work was the most likely hypothesis proposed by the authors to explain the acquisition of ST8 infection by zookeepers. Moreover, *Blastocystis* sp. isolated from zookeepers at Perth Zoo, Australia, were also found to be identical to some isolates from NHPs, which are clustered in the same ST1 [23]. Although the risk of transmission of the parasite from NHPs to humans seems well evidenced, it is probably rather limited, because these two host groups are living in different ecological niches, strongly preventing possible contact. Consequently, the zoonotic transmission of *Blastocystis* sp. from NHPs may occur mainly in zoos and animal sanctuaries, as suggested previously [32].

**Table 3:** Compilation of data in both the present study and in the literature at the time of publication relative to the *Blastocystis* sp. STs identified in different animal groups worldwide (modified and completed from Stensvold et al. [24] and Alfellani et al. [32]).

Host	*Blastocystis* sp. STs																		Mixed ST/ Untypable	Ref.
	1	2	3	4	5	6	7	8	9	10	11	12	13	14	15	16	17	NMASTs^a^		
*EUTHERIA*																				
**HUMANS (total)**	**882**	**343**	**1399**	**318**	**9**	**89**	**118**	**10**	**3**	**0**	**0**	**0**	**0**	**0**	**0**	**0**	**0**	**0**	**225**	[19]
NON-HUMAN PRIMATES																				
Hominidae	34	27	27	0	18	0	0	0	0	0	0	0	0	0	1	0	0	0	4	[32]
	13	10	0	0	0	0	0	0	0	0	7	0	0	0	0	0	0	0	4	[33]
	1	2	0	0	6	0	0	0	0	0	0	0	0	0	0	0	0	0	0	**Present study**
Hylobatidae	12	8	3	0	2	0	0	7	0	0	0	0	0	0	1	0	0	0	0	[32]
	2	1	3	1	0	0	0	0	0	0	0	0	0	0	1	0	0	0	0	**Present study**
Cercopithecidae	76	48	73	0	4	0	0	1	0	0	0	0	5	0	0	0	0	0	19	[32]
	5	0	1	0	0	0	0	0	0	0	0	0	0	0	0	0	0	0	0	[33]
	54	67	33	0	1	0	34	0	0	0	0	0	0	0	0	0	0	0	0	[43]
	7	2	4	0	0	0	0	0	0	0	0	0	0	0	0	0	0	0	0	**Present study**
Cebidae	10	7	12	1	0	0	0	24	0	0	0	0	0	0	0	0	0	0	1	[32]
	3	0	1	0	0	0	0	0	0	0	0	0	0	0	0	0	0	0	0	**Present study**
Lemuridae	4	2	0	4	0	0	0	3	0	2	0	0	0	0	1	0	0	0	0	[32]
	2	3	0	3	1	0	2	2	0	0	0	0	0	0	0	0	0	0	0	**Present study**
Others^b^	0	0	0	2	0	0	0	0	0	0	0	0	0	0	0	0	0	0	0	[6]
	0	0	0	0	0	0	0	47	0	0	0	0	0	0	0	0	0	0	0	[44]
	0	1	0	0	0	0	0	0	0	0	0	0	0	0	0	0	0	0	0	**Present study**
**Total**	**223**	**178**	**157**	**11**	**32**	**0**	**36**	**84**	**0**	**2**	**7**	**0**	**5**	**0**	**4**	**0**	**0**	**0**	**28**	
CARNIVORA																				
	8	3	6	3	0	0	3	0	0	0	0	0	0	0	0	0	0	0	0	[19]
	11	1	0	2	1	7	0	0	0	0	0	0	0	0	0	0	0	0	0	[51]
	0	0	8	0	0	0	0	0	0	0	0	0	0	0	0	0	0	0	0	[70]
	0	15	0	0	0	0	0	0	0	0	0	0	0	0	0	0	0	0	0	[6]
	3	0	0	0	0	0	0	0	0	6	0	0	0	2	0	0	0	0	0	[52]
	0	2	0	0	0	0	0	0	0	2	0	0	0	0	0	0	0	0	0	[37]
	1	2	4	3	3	0	0	0	0	0	0	0	0	0	0	0	0	0	10	[54]
	1	1	2	0	0	0	0	0	0	0	0	0	0	0	0	0	0	0	0	**Present study**
**Total**	**24**	**24**	**20**	**8**	**4**	**7**	**3**	**0**	**0**	**8**	**0**	**0**	**0**	**2**	**0**	**0**	**0**	**0**	**10**	
ARTIODACTYLA																				
Bovidae	2	0	4	0	12	0	0	0	0	23	0	0	0	0	0	0	0	0	0	[24]
	0	0	0	0	0	0	0	0	0	7	0	0	0	0	0	0	0	0	0	[26]
	0	0	0	0	0	2	0	0	0	0	0	0	0	0	0	0	0	0	4	[28]
	0	0	0	0	0	0	0	0	0	8	0	0	0	3	0	0	0	0	0	[36]
	44	0	8	0	0	30	30	0	0	0	0	0	0	0	0	0	0	0	0	[55]
	1	0	1	0	3	0	1	0	0	19	0	0	0	3	1	0	0	0	14	[19]
	12	0	8	0	0	0	0	0	0	0	0	0	0	0	0	0	0	0	0	[6]
	0	0	4	0	11	2	0	0	0	0	0	0	0	0	0	0	0	0	4	[56]
	0	0	1	0	0	0	0	0	0	12	0	0	0	4	0	0	0	0	0	**Present study**
Subtotal	**59**	**0**	**26**	**0**	**26**	**34**	**31**	**0**	**0**	**69**	**0**	**0**	**0**	**10**	**1**	**0**	**0**	**0**	**22**	
Suidae	150	7	6	0	54	0	0	0	0	0	0	0	0	0	0	0	0	0	0	[24]
	0	0	0	0	7	0	0	0	0	0	0	0	0	0	0	0	0	0	0	[26]
	0	0	0	0	0	2	0	0	0	0	0	0	0	0	0	0	0	0	2	[28]
	3	0	2	0	8	0	0	0	0	0	0	0	0	0	0	0	0	0	0	[33]
	0	0	0	0	13	0	0	0	0	0	0	0	0	0	0	0	0	0	0	[19]
	0	0	0	0	5	0	0	0	0	0	0	0	0	0	0	0	0	0	0	[71]
	9	0	7	0	217	0	0	0	0	0	0	0	0	0	0	0	0	0	17	[30]
	43	39	0	0	73	0	8	0	0	0	0	0	0	0	0	0	0	0	0	[57]
Subtotal	**205**	**46**	**15**	**0**	**377**	**2**	**8**	**0**	**0**	**0**	**0**	**0**	**0**	**0**	**0**	**0**	**0**	**0**	**19**	
Others^c^	0	0	0	0	0	0	0	0	0	1	0	0	0	0	0	0	0	0	0	[24]
	0	0	0	0	0	0	0	0	0	0	0	4	0	0	0	0	0	0	0	[25]
	0	0	0	1	0	0	0	0	0	0	0	1	0	0	0	0	0	0	0	[33]
	5	0	6	0	21	0	0	0	0	8	0	0	2	3	1	0	0	0	7	[19]
	0	0	0	0	0	0	0	0	0	2	0	0	1	4	0	0	0	0	0	**Present study**
**Total**	**269**	**46**	**47**	**1**	**424**	**36**	**39**	**0**	**0**	**80**	**0**	**5**	**3**	**17**	**2**	**0**	**0**	**0**	**48**	
PERISSOSACTYLA																				
	1	0	0	0	1	0	0	0	0	0	0	0	0	0	0	0	0	0	0	[19]
	0	1	5	0	1	0	0	0	0	0	0	0	0	0	0	0	0	0	1	**Present study**
**Total**	**1**	**1**	**5**	**0**	**2**	**0**	**0**	**0**	**0**	**0**	**0**	**0**	**0**	**0**	**0**	**0**	**0**	**0**	**1**	
PROBOSCIDEA																				
	0	0	0	0	0	0	0	0	0	0	0	0	0	0	15	0	0	0	0	[19]
	3	0	2	0	0	0	0	0	0	0	0	0	0	0	0	0	0	0	0	**Present study**
**Total**	**3**	**0**	**2**	**0**	**0**	**0**	**0**	**0**	**0**	**0**	**0**	**0**	**0**	**0**	**15**	**0**	**0**	**0**	**0**	
RODENTIA																				
	0	0	4	7	1	0	0	0	0	0	0	0	0	0	0	0	1	0	0	[19]
	0	3	0	0	0	0	0	0	0	0	0	0	0	0	0	0	0	0	0	[6]
	0	0	0	9	0	0	0	0	0	0	0	0	0	0	0	0	0	0	0	[57]
	0	1	0	1	1	0	0	0	0	0	0	0	0	0	0	0	0	0	1	**Present study**
**Total**	**0**	**4**	**4**	**17**	**2**	**0**	**0**	**0**	**0**	**0**	**0**	**0**	**0**	**0**	**0**	**0**	**1**	**0**	**1**	
CHIROPTERA	**0**	**0**	**2**	**0**	**0**	**0**	**0**	**0**	**0**	**0**	**0**	**0**	**0**	**0**	**0**	**0**	**0**	**0**	**0**	**Present study**
*MARSUPIALIA*																				
	0	0	0	10	0	0	0	0	0	0	0	2	3	0	0	2	0	0	0	[19]
	0	0	0	0	0	0	0	25	0	0	0	0	0	0	0	0	0	0	0	[6]
	0	0	0	0	0	0	0	0	0	0	0	0	0	0	0	0	0	0	1	**Present study**
**Total**	**0**	**0**	**0**	**10**	**0**	**0**	**0**	**25**	**0**	**0**	**0**	**2**	**3**	**0**	**0**	**2**	**0**	**0**	**1**	
*AVES*																				
Anseriformes	0	0	0	0	0	0	2	0	0	0	0	0	0	0	0	0	0	0	0	[19]
Galliformes	3	2	0	0	0	11	15	0	0	0	0	0	0	0	0	0	0	0	7	[19]
	0	0	0	0	0	0	8	0	0	0	0	0	0	0	0	0	0	0	0	[57]
	0	0	0	0	0	0	0	0	0	0	0	0	0	0	0	0	0	0	1	**Present study**
Passeriformes	0	0	0	0	0	0	47	0	0	0	0	0	0	0	0	0	0	0	0	[6]
Others^d^	1	0	0	0	0	0	0	0	0	0	0	0	0	0	0	0	0	0	0	**Present study**
Ratites	0	2	0	6	0	0	0	0	0	0	0	0	0	0	0	0	0	0	0	[19]
	0	0	0	0	0	14	0	0	0	0	0	0	0	0	0	0	0	0	23	[63]
	0	0	0	2	3	0	0	0	0	0	0	0	0	0	0	0	0	0	0	**Present study**
Unidentified	2	0	0	0	0	0	3	0	0	0	0	0	0	0	0	0	0	0	0	[19]
**Total**	**6**	**4**	**0**	**8**	**3**	**25**	**75**	**0**	**0**	**0**	**0**	**0**	**0**	**0**	**0**	**0**	**0**	**0**	**31**	
*SQUAMATA*																				
	0	0	0	0	0	0	0	0	0	0	0	0	0	0	0	0	0	4	0	[67]
	0	0	0	0	0	0	0	0	0	0	0	0	0	0	0	0	0	3	0	[20]
	0	0	0	0	0	0	0	0	0	0	0	0	0	0	0	0	0	0	2	**Present study**
**Total**	**0**	**0**	**0**	**0**	**0**	**0**	**0**	**0**	**0**	**0**	**0**	**0**	**0**	**0**	**0**	**0**	**0**	**7**	**2**	
*TESTUDINATA*																				
	0	0	0	0	0	0	0	0	0	0	0	0	0	0	0	0	0	5	0	[68]
	0	0	0	0	0	0	0	0	0	0	0	0	0	0	0	0	0	1	0	[67]
	0	0	0	0	0	0	0	0	0	0	0	0	0	0	0	0	0	11	0	[20]
	0	0	0	0	0	0	0	0	0	0	0	0	0	0	0	0	0	3	0	**Present study**
**Total**	**0**	**0**	**0**	**0**	**0**	**0**	**0**	**0**	**0**	**0**	**0**	**0**	**0**	**0**	**0**	**0**	**0**	**20**	**0**	
*AMPHIBIA*	**0**	**0**	**0**	**0**	**1**	**0**	**0**	**0**	**0**	**0**	**0**	**0**	**0**	**0**	**0**	**0**	**0**	**3**	**0**	[67]
*INSECTA*	0	0	0	0	0	0	0	0	0	0	0	0	0	0	0	0	0	4	0	[72]
	0	2	1	0	0	0	0	0	0	0	0	0	0	0	0	0	0	0	0	**Present study**
**Total**	**0**	**2**	**1**	**0**	**0**	**0**	**0**	**0**	**0**	**0**	**0**	**0**	**0**	**0**	**0**	**0**	**0**	**4**	**0**	
**Grand total excluding humans**	**526**	**259**	**238**	**55**	**468**	**68**	**153**	**109**	**0**	**90**	**7**	**7**	**11**	**19**	**21**	**2**	**1**	**34**	**122**	

^a^ Non Mammalian and Avian STs

^b^ Including Atelidae and Pitheciidae

^c^ Including Tragulidae, Giraffidae and Camelidae

^d^ Including Phoenicopteriformes

The group of carnivores is of great interest since it covers most pets (domestic cats and dogs) that, due to their close contact with humans, may represent a potential source of zoonotic transmission of *Blastocystis* sp. The four isolates from carnivores identified in French zoos belonged to ST1, ST2 and ST3 (two isolates) (Table 2) and their sequences showed 100% identity with those of humans, with the exception of one ST3 isolate whose sequence was shown to be 99% similar. Interestingly, these three STs are predominant in this animal group according to the data compiled in previous studies (Table 3) as well as in humans, suggesting possible cross-transmission between these hosts. However, to our knowledge, only three recent papers focusing on domestic dogs and their owners in various countries showed a potential relationship between carnivore carriers and human infection through ST concordance [29,54,74]. In addition, the low to moderate prevalence of *Blastocystis* sp. observed in numerous canine cohorts [37,49,51,52,54], together with the absence of a dog-specific/predominant ST, strongly suggest that dogs are unlikely to be natural hosts of *Blastocystis* sp. The recent study by Wang et al. [51] confirmed this hypothesis by demonstrating that stray dogs in India showed both greater diversity of STs and a higher prevalence of the parasite than domestic dogs in Australia and Cambodia. For the authors, dogs may be transiently and opportunistically infected by *Blastocystis* sp. via coprophagia, and the higher prevalence and larger ST diversity observed in stray dogs compared to domestic dogs could be attributed to greater exposure to fecal material from human and non-human hosts in their environment. Consequently, and in agreement with the present data, carnivores and especially Canidae do not seem to represent a significant source of *Blastocystis* sp. infection in humans [37,51].

Regarding artiodactyls, representatives of 5 families were sampled in our survey. Of the 21 samples sequence-positive for *Blastocystis* sp. by qPCR in this animal group, three presented mixed infections by two different STs (all with ST10 and ST14). Therefore, a total of 24 isolates were subtyped, and more than half of them (58.3%) belonged to ST10 (Table 2). The remaining isolates were identified as ST14 (33.3%), ST13 (4.2%) and ST3 (4.2%). Interestingly, three of the four families of Artiodactyla screened in our study (Camelidae, Giraffidae and Bovidae) were infected by ST10 and/or ST14. The fourth family, Tragulidae, was represented by a single species (Java mouse-deer) at the Lille Zoo, which was infected by ST13. However, this very rare ST was also described in a mouse deer from UK [19]. Strikingly, the distribution of STs in Bovidae in the present study was highly similar to that obtained by Fayer et al. [36] from cattle feces in the USA, in which all isolates identified belonged to either ST10 or ST14. Cattle from the USA were also shown to be infected only with ST10 [26], which was also the predominant ST in cohorts of cattle in Denmark [24], the UK and Libya [19]. All this worldwide data, together with those of the current study, strongly suggest that Bovidae may be natural hosts of *Blastocystis* sp. ST10 and ST14 (Table 3). Despite common exposure of humans to bovids, for instance livestock, both STs have never yet been reported in humans (Fig 2). In addition to ST10 and ST14, ST1 was also described in bovids, especially in goats [55] and cattle [6] with high prevalence (Table 3), but the search for the potential transmission of bovid ST1 isolates to human remains poorly documented. Still among artiodactyls, representatives of Suidae could not be sampled in the present study, because they are not housed in the two French zoos. However, this animal group was shown to be frequently infected, primarily by ST1 and ST5, suggesting that pigs are likely to be natural hosts of these *Blastocystis* sp. STs [24,30,35,57] (Table 3). The potential of pigs to act as zoonotic reservoirs was recently demonstrated by Wang et al. [30], who found an unusually high prevalence of ST5 in both pigs and piggery staff in Australia, as well as sequence identity of ST1 and ST5 isolates from pigs and piggery workers. Therefore, close contact between pigs and their handlers may clearly increase the risk of zoonotic transmission of *Blastocystis* sp. ST1 and ST5 (Fig 2).

For other mammalian groups, the current data are too limited to draw conclusions about a potential risk of zoonotic transmission, and the present study only reinforces the currently available databases. In Perissodactyla, since 1 mixed infection by 2 STs was identified from a Poitou donkey, 8 isolates in total have been subtyped, and mostly belonged to ST3 (5/8 isolates) (Table 2). One isolate belonging to ST2 was also identified in the Poitou donkey, as well as one isolate described as ST5 in the South American tapir. A last isolate was assigned as untypable in the common zebra (isolate ZLB10) (Fig 1). In previous studies, the only two Perissodactyla isolates subtyped so far in a black rhinoceros and a horse corresponded to ST5 and ST1 sequences respectively [19] (Table 3). In Proboscidea, 5 isolates were subtyped from Asiatic elephants, since two samples presented mixed infections by two STs. All the isolates belonged to ST1 or ST3 (Table 2), while previous isolates identified and subtyped from captive elephants housed in Australian and European zoos or wild animals in Australia were all shown to belong to ST11 [25,33] (Table 3). In rodents, 4 isolates were subtyped in the present study and three of them belonged to ST2, ST4 and ST5, respectively. A last isolate was assigned as untypable (isolate LPA10) (Table 2 and Fig 1). Interestingly, ST2 was previously identified in wild rats in Colombia [6], while ST4 was the only ST identified in wild rats in Indonesia [55]. Rodents were previously proposed to be a reservoir of ST4, but the present study, as well as the study by Alfellani et al. [19], confirmed that other STs can be found in this host group. Regarding bats, two ST3 isolates were identified in the Rodrigues flying fox and Egyptian fruit bat (Table 2), both belonging to Pteropotidae and housed at the La Palmyre Zoo. To our knowledge, this is the first identification of *Blastocystis* sp. in Chiroptera. Among marsupials, one of the two samples tested was shown to be infected by an isolate for the time being classified as untypable. Only a few previous surveys reported ST4, ST8, ST12,ST13 and ST16 in this animal group [6,24,25,33,47] (Table 3). Overall, more intensive sampling should be performed in these five as yet underrepresented mammalian groups, with the aim of confirming that each of them represents the natural host of the parasite and, if so, to assign a specific ST to these hosts.

Regarding non-mammalian groups, birds have already been considered as potential reservoirs of *Blastocystis* sp. transmission to humans [19,22,24]. Indeed, birds usually host ST6 and ST7, which are considered “avian STs” because of their predominance in this host group (about 82% of all avian isolates subtyped so far), as shown in Table 3. Thus, these two rare STs in humans, accounting for around 9% of the human samples across the world according to Alfellani et al. [21], were suggested to be of zoonotic origin, as supported by the high similarity or even identity of SSU rRNA gene sequences between human and avian isolates [22] (Fig 2). Nevertheless, of the 6 avian isolates characterized in the present study, none were identified as belonging to the “avian” ST6 or ST7. Three of them belonged to ST5, two to ST4, one to ST1, and the last one to NMAST (Table 2), suggesting that other STs can occasionally infect this host group. As stated above, the prevalence of *Blastocystis* sp. was very low among birds in both French zoos, which could easily be explained by the daily cleaning of cages and the small number of animals per cage. On the other hand, the prevalence of the parasite was shown to be very high in poultry flocks on commercial farms, due to high population density [60,61]. Therefore, to clarify the impact of zoonotic transmission from poultry, an epidemiological survey should be conducted promptly among the staff of these commercial farms who work in close contact with animals.

Among reptiles, 1 mixed infection by 2 STs was found in a spur-thighed tortoise. A total of 5 isolates were thus subtyped from this animal group (Table 2). Three of them were shown to belong to various NMASTs, while the other two isolates were classified as untypable (Fig 1). Interestingly, all sequences obtained so far from reptilian isolates were also derived from NMASTs (Table 3). This strongly suggested that NMASTs could be specific to this animal group and are only occasionally found in other hosts, including mammals through accidental contamination as hypothesized in the present study for several species.

Finally, the 3 insect isolates subtyped in the present study belonged to ST2 (2 isolates) and ST3 (Table 2), which are commonly found STs in other animal groups and humans (Table 3). In addition, the three isolates showed 98 to 99% identity with human and various animal isolates, suggesting accidental contamination of insects by animal or human feces. Indeed, these subtyping data strongly contrast with those obtained previously by Yoshikawa et al. [58] regarding four *Blastocystis* sp. isolates identified in cockroaches and belonging to NMAST (Fig 1). As very little data are currently available for insects, the predominance of NMAST in this animal group remains to be confirmed by sampling additional taxonomic groups of this diversified class of organisms.

## Conclusions

To our knowledge, the present study represents the widest epidemiological survey ever conducted to increase our knowledge of the role of various animal groups in the epidemiology of *Blastocystis* sp. Overall the parasite was commonly identified in animals housed in two French zoos, with varying prevalence from one host to another. Even though the data were generated from zoo animals and should be interpreted with caution, the findings in widely sampled groups including NHPs showed a roughly similar ST distribution in captive and wild animals. By combining our molecular data with data obtained in previous surveys and comparing the summarized overall ST distribution between animals and humans, it appears that NHPs and artiodactyls, especially livestock, could represent potential reservoirs of zoonotic transmission of *Blastocystis* sp. (Fig 2). Regarding other mammalian groups such as carnivores, perissodactyls, proboscids, rodents, bats and marsupials, their lower sampling currently prevents any assignment of host-specific STs and the evaluation of zoonotic risk, while knowing that close intimate association with animals increases the risk of acquiring *Blastocystis* sp. infection. Moreover, since most of these mammalian groups were shown to be frequently infected by STs commonly found in humans, the direction of transmission cannot be established with any certainty. In the case of non-mammalian groups, insects and reptiles are generally infected by NMASTs that are only found accidentally in other animal groups. Moreover, even if *Blastocystis* sp. were sporadically identified in birds in both French zoos, the high prevalence of the parasite in poultry from commercial farms, combined with the avian specificity of ST6 and ST7 and the identification of both STs in humans, strongly suggest that birds could play a significant role as a reservoir for zoonotic transmission (Fig 2). These data also stress the importance of screening other hosts of *Blastocystis* sp. in order to complete the epidemiology of this parasite. For instance, a single study dating back over 20 years identified the parasite in fish [75], and the detection of *Blastocystis* sp. was only recently described in marine mollusks [76]. Even if these surveys extend the known host range of the parasite, molecular data is lacking for these hosts, although they could represent potential sources of zoonotic transmission through their raw consumption and / or handling. Finally, this epidemiological survey also provides necessary information for taking preventive and control measures that should help to reduce the burden of *Blastocystis* spp. in zoos and the risks of zoonotic transmission to animal handlers.

## Supporting Information

S1 Table Isolation source (center), season of collect, ST identification and GenBank accession number of *Blastocystis* sp. isolates characterized in our study. (DOCX)
