# Supplementary material for: Molecular Epidemiology of Blastocystis sp. in Various Animal Groups from Two French Zoos and Evaluation of Potential Zoonotic Risk
Source: PLoS One. 2017 Jan 6;12(1):e0169659. doi: 10.1371/journal.pone.0169659 (PMC5217969; doi:10.1371/journal.pone.0169659)
Supplement: S1 Table — (DOCX) [file pone.0169659.s001.docx]

**Table S1. Isolation source (zoo), animal host, ST identification and GenBank accession number of *Blastocystis* sp. isolates characterized in our study.**

| Isolate | Zoo^a^ | Animal host | *Blastocystis* sp. ST | GenBank accession number |
| --- | --- | --- | --- | --- |
| LPP1 | LP | *Gorilla gorilla* | ST2 | KR259402 |
| LPP2 | LP | *Gorilla gorilla* | ST1 | KR259403 |
| LPP3 | LP | *Gorilla gorilla* | ST5 | KR259404 |
| LPP4 | LP | *Gorilla gorilla* | ST5 | KR259405 |
| LPP7 | LP | *Pongo pygmaeus* | ST5 | KR259406 |
| LPP8 | LP | *Pongo pygmaeus* | ST5 | KR259407 |
| LPP9 | LP | *Pongo pygmaeus* | ST5 | KR259408 |
| LPP10 | LP | *Pan troglodytes* | ST2 | KR259409 |
| LPP23 | LP | *Pan troglodytes* | ST5 | KR259410 |
| LPP16 | LP | *Nomascus gabriellae* | ST1 | KR259411 |
| LPP17 | LP | *Nomascus gabriellae* | ST1 | KR259412 |
| LPP13 clone 1 | LP | *Macaca nemestrina* | ST3 | KR259413 |
| LPP13 clone 2 | LP | *Macaca nemestrina* | ST1 | KR259414 |
| LPP14 | LP | *Macaca nemestrina* | ST1 | KR259415 |
| LPP15 | LP | *Macaca nemestrina* | ST1 | KR259416 |
| LPP18 | LP | *Mandrillus sphinx* | ST1 | KR259417 |
| LPP56 | LP | *Cercopithecus hamlyni* | ST1 | KR259418 |
| LPP31 | LP | *Cercopithecus roloway* | ST2 | KR259419 |
| LPP35 | LP | *Cercopithecus lhoesti* | ST3 | KR259420 |
| LPP36 clone 1 | LP | *Cercopithecus neglectus* | ST3 | KR259421 |
| LPP36 clone 2 | LP | *Cercopithecus neglectus* | ST1 | KR259422 |
| LPP37 | LP | *Cercopithecus neglectus* | ST3 | KR259423 |
| LPP54 | LP | *Colobus guereza* | ST2 | KR259424 |
| LPP55 | LP | *Colobus guereza* | ST1 | KR259425 |
| LPP39 | LP | *Saguinus imperator* | ST3 | KR259426 |
| LPP42 | LP | *Leonpithecus chrysomelas* | ST1 | KR259427 |
| LPP43 | LP | *Leonpithecus chrysomelas* | ST1 | KR259428 |
| LPP44 | LP | *Leonpithecus chrysomelas* | ST1 | KR259429 |
| LPP21 | LP | *Lemur catta* | ST2 | KR259430 |
| LPP22 | LP | *Lemur catta* | ST2 | KR259431 |
| LPP26 | LP | *Lemur catta* | ST2 | KR259432 |
| LPP45 | LP | *Lemur catta* | ST1 | KR259433 |
| LPP19 clone 1 | LP | *Varecia rubra* | ST4 | KR259434 |
| LPP19 clone 2 | LP | *Varecia rubra* | ST8 | KR259435 |
| LPP19 clone 3 | LP | *Varecia rubra* | ST1 | KR259436 |
| LPP24 | LP | *Varecia rubra* | ST5 | KR259437 |
| LPP25 | LP | *Varecia variegata* | ST4 | KR259438 |
| LPP52 | LP | *Eulemur flavifrons* | ST4 | KR259439 |
| LPC7 | LP | *Acinonyx jubatus* | ST2 | KR259440 |
| LPC11 | LP | *Canis lupus* | ST3 | KR259441 |
| LPC3 | LP | *Ursus maritimus* | ST3 | KR259442 |
| LPC41 | LP | *Zalophus californianus* | ST1 | KR259443 |
| LPM9 clone 1 | LP | *Giraffa camelopardalis* | ST14 | KR259444 |
| LPM9 clone 2 | LP | *Giraffa camelopardalis* | ST10 | KR259445 |
| LPM10 | LP | *Giraffa camelopardalis* | ST14 | KR259446 |
| LPM11 | LP | *Giraffa camelopardalis* | ST14 | KR259447 |
| LPM13 | LP | *Giraffa camelopardalis* | ST14 | KR259448 |
| LPM20 | LP | *Tragelaphus strepsiceros* | ST10 | KR259449 |
| LPM21 | LP | *Tragelaphus strepsiceros* | ST10 | KR259450 |
| LPM22 | LP | *Tragelaphus eurycerus* | ST10 | KR259451 |
| LPM44 clone 1 | LP | *Bison bison* | ST14 | KR259452 |
| LPM44 clone 2 | LP | *Bison bison* | ST10 | KR259453 |
| LPM23 | LP | *Connochaetes taurinus* | ST14 | KR259454 |
| LPM33 | LP | *Oryx beisa* | ST10 | KR259455 |
| LPM34 | LP | *Oryx beisa* | ST10 | KR259456 |
| LPM35 | LP | *Oryx beisa* | ST10 | KR259457 |
| LPM36 | LP | *Oryx dammah* | ST10 | KR259458 |
| LPM37 | LP | *Oryx dammah* | ST10 | KR259459 |
| LPM38 | LP | *Oryx dammah* | ST10 | KR259460 |
| LPM39 | LP | *Oryx dammah* | ST10 | KR259461 |
| LPM40 | LP | *Oryx dammah* | ST3 | KR259462 |
| LPM8 clone 1 | LP | *Capra hircus* | ST10 | KR259463 |
| LPM8 clone 2 | LP | *Capra hircus* | ST14 | KR259464 |
| LPM25 | LP | *Equus burchelii* | ST3 | KR259465 |
| LPM29 | LP | *Equus asinus* | ST3 | KR259466 |
| LPM30 clone 1 | LP | *Equus asinus* | ST2 | KR259467 |
| LPM30 clone 2 | LP | *Equus asinus* | ST3 | KR259468 |
| LPM31 | LP | *Tapirus terrestris* | ST3 | KR259469 |
| LPM32 | LP | *Tapirus terrestris* | ST3 | KR259470 |
| LPA4 | LP | *Elephas maximus* | ST1 | KR259471 |
| LPA5 clone 1 | LP | *Elephas maximus* | ST3 | KR259472 |
| LPA5 clone 2 | LP | *Elephas maximus* | ST1 | KR259473 |
| LPA6 clone 1 | LP | *Elephas maximus* | ST1 | KR259474 |
| LPA6 clone 2 | LP | *Elephas maximus* | ST3 | KR259475 |
| LPA8 | LP | *Hydrochoerus hydrochaeris* | ST2 | KR259476 |
| LPA10 | LP | *Hydrochoerus hydrochaeris* | Untypable | KR259477 |
| LPA1-1 | LP | *Pteropus rodricensis* | ST3 | KR259478 |
| LPA1-2 | LP | *Rousettus aegyptiacus* | ST3 | KR259479 |
| LPA3 | LP | *Macropus rufogriseus* | Untypable | KR259480 |
| LPO12 | LP | *Pavo cristatus* | Untypable | KR259481 |
| LPO19 | LP | *Phoenicopterus ruber* | ST1 | KR259482 |
| LPO35 | LP | *Struthio camelus* | ST5 | KR259483 |
| LPO36 | LP | *Struthio camelus* | ST5 | KR259484 |
| LPO38 | LP | *Rhea americana* | ST4 | KR259485 |
| LPO39 clone 1 | LP | *Rhea americana* | ST5 | KR259486 |
| LPO39 clone 2 | LP | *Rhea americana* | ST4 | KR259487 |
| ZLA1 | ZL | *Varecia rubra* | ST7 | KR259488 |
| ZLA2 | ZL | *Varecia variegata* | ST7 | KR259489 |
| ZLA3 | ZL | *Lemur catta* | ST8 | KR259490 |
| ZLA15 | ZL | *Pithecia pithecia* | ST2 | KR259491 |
| ZLA19 | ZL | *Rattus norvegicus* | ST4 | KR259492 |
| ZLA31 | ZL | *Blaberus giganteus* | ST2 | KR259493 |
| ZLA33-3 | ZL | *Blaptica dubia* | ST3 | KR259494 |
| ZLA35-2 | ZL | *Schistocerca gregaria* | ST2 | KR259495 |
| ZLB5 | ZL | *Hydrochoerus hydrochaeris* | ST5 | KR259496 |
| ZLB6 | ZL | *Vicugna pacos* | ST10 | KR259497 |
| ZLB10 | ZL | *Equus burchelii* | Untypable | KR259498 |
| ZLB12 | ZL | *Taurotragus oryx* | ST14 | KR259499 |
| ZLB15 | ZL | *Symphalangus syndactylus* | ST3 | KR259500 |
| ZLB16 | ZL | *Symphalangus syndactylus* | ST4 | KR259501 |
| ZLB17 | ZL | *Symphalangus syndactylus* | ST3 | KR259502 |
| ZLB18 | ZL | *Symphalangus syndactylus* | ST3 | KR259503 |
| ZLB22 | ZL | *Tapirus terrestris* | ST5 | KR259504 |
| ZLB25 | ZL | *Hylobates lar* | ST15 | KR259505 |
| ZLB26 | ZL | *Hylobates lar* | ST2 | KR259506 |
| ZLB27 | ZL | *Aldabrachelys gigantea* | NMAST^b^ | KR259507 |
| ZLB30 | ZL | *Iguana iguana* | Untypable | KR259508 |
| ZLB37 | ZL | *Tragulus javanicus* | ST13 | KR259509 |
| ZLC1 clone 1 | ZL | *Testudo graeca* | NMAST | KR259510 |
| ZLC1 clone 2 | ZL | *Testudo graeca* | NMAST | KR259511 |
| ZLC7 | ZL | *Boa constrictor* | Untypable | KR259512 |

^a^ ZL : Zoo of Lille ; LP : Zoo of La Palmyre

^b^ NMAST: Non Mammalian and Avian ST
